# Regulation mechanism of ferroptosis and its research progress in tumor immunotherapy

**DOI:** 10.3389/fmolb.2022.1045548

**Published:** 2022-10-25

**Authors:** Jing Xu, Xiaolin Lin, Ting Han, Qingqing Zhou, Yang Su, Shuqing Jiang, Xiuying Xiao, Tengfei Liu

**Affiliations:** ^1^ Jining Medical University, Jining, China; ^2^ Department of Oncology, Renji Hospital, Shanghai Jiao Tong University School of Medicine, Shanghai, China; ^3^ Department of Oncology, Ruijin Hospital, Shanghai Jiao Tong University School of Medicine, Shanghai, China; ^4^ State Key Laboratory of Oncogenes and Related Genes, Shanghai Cancer Institute, Renji Hospital, Shanghai Jiao Tong University School of Medicine, Shanghai, China

**Keywords:** ferroptosis, anti-tumor immunotherapy, review, ferroptosis inducer and inhibitor, metabolic pathway

## Abstract

Ferroptosis is a novel regulatory cell death, which is characterized by iron dependency and mainly caused by accumulation of intracellular lipid peroxides and reactive oxygen species. Ferroptosis plays an important role in the occurrence and development of a variety of malignant tumors, especially in anti-tumor treatment. As an emerging treatment method, the immunotherapy has been widely applied in the clinical practice, and the role of ferroptosis in tumor immunotherapy has been gradually explored. This study aims to illustrate the features of ferroptosis, and its role in anti-tumor immunotherapy and potential clinical application.

## Introduction

Ferroptosis, as a new form of cell death, plays an important role in many diseases, especially affecting the malignant progress of tumors and anti-tumor treatment (Liang et al., 2019; Chen et al., 2021a; Wu et al., 2020). Anti-tumor treatment is divided into drug therapy, radiation therapy, surgical treatment and so on, among which drug therapy includes immunotherapy, chemotherapy, targeted therapy, etc. Among them, anti-tumor immunotherapy, such as targeting PD-1 (programmed death-1 protein-1 (PD-1) or its ligand PD-L1 or CTLA4, aims to strengthen the immune system and exerts anti-tumor effects. Immunotherapy has been increasingly applied in the treatment of various malignant tumors, and exhibits well therapeutic effect and long-term benefit, while its curative evaluation still remains unclear. Studies have shown that targeting ferroptosis is expected to improve the therapeutic effect of anti-tumor immunotherapy, suggesting the potential relationship between ferroptosis and immunotherapy. Therefore, this review intends to summarize the role and research progress of ferroptosis in anti-tumor immunotherapy, and provide reference and hints for follow-up research.

## Overview and characteristics of ferroptosis

**FIGURE 1:**
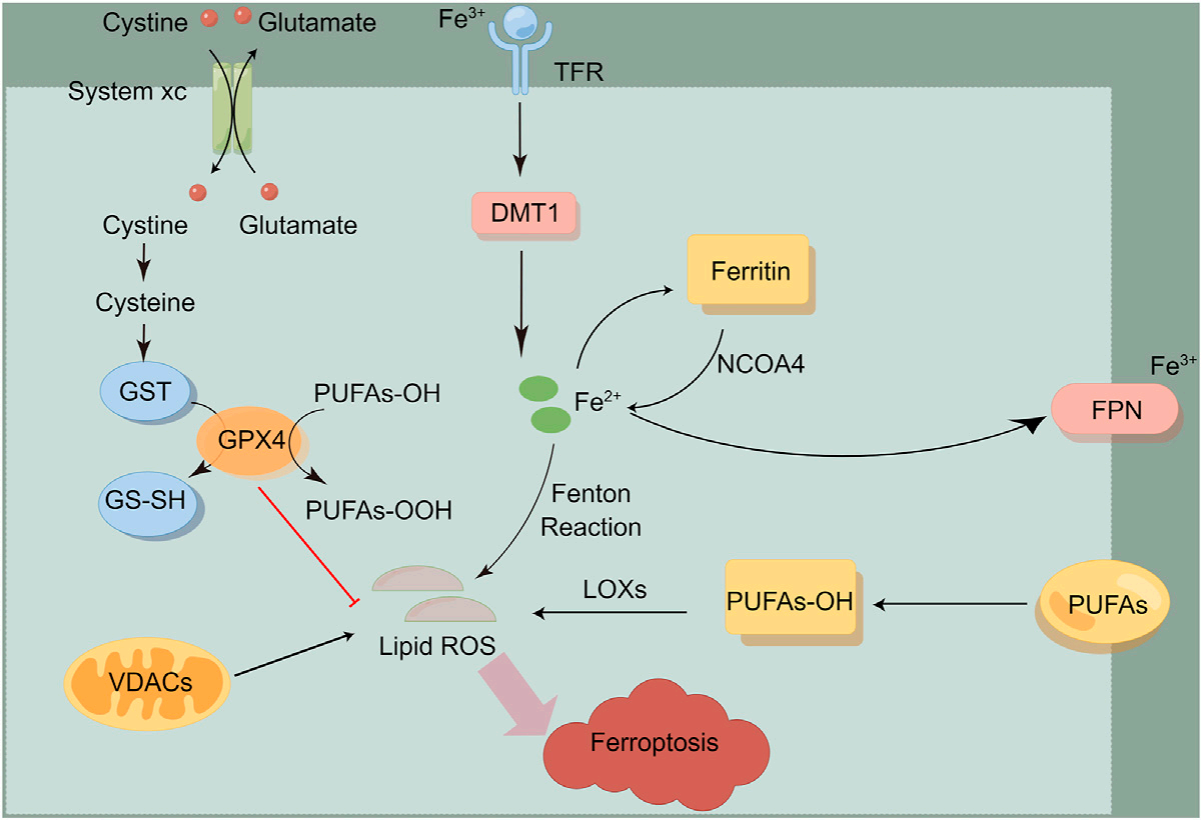
The overview of ferroptosis. Ferroptosis is mainly induced by iron metabolism, lipid metabolism, amino acid metabolism and other processes. This figure was drawn by Figdraw.

The concept of “ferroptosis” was firstly put forward by Dixon in 2012 (Dixon et al., 2012) (Figure 1). It is a form of iron-dependent cell death, characterized by excessive oxidative stress and membrane lipid peroxidation. Ferroptosis is different from other types of cell death (such as apoptosis, necrosis, etc.) in morphological changes, biochemical characteristics and regulatory mechanisms (Chen et al., 2021). As for morphology, cell apoptosis is characterized by intact cell membrane, shrinking chromatin, and the formation of symbolic apoptotic bodies. Cell necrosis and pyroptosis showed obvious cell swelling and moderate chromatin concentration, while ferroptosis had no obvious apoptotic characteristics, mainly showing smaller mitochondria, decreased mitochondrial cristae, and increased mitochondrial membrane density. As for ferroptosis, the ultrastructural changes of mitochondrial membrane concentration and rupture are considered as the unique morphological signs (Battaglia et al., 2020).

## Inducer and inhibitor of ferroptosis

In recent years, an increasing numbers of natural and synthetic drugs related to ferroptosis have been identified, including inducers and inhibitors. The rational way to apply has become an important proposition to improve anti-tumor effectiveness. According to the mechanism of ferroptosis, Doxin et al. divided ferroptosis inducers (FINS) into four categories: targeting system Xc-, GPX4, iron and ROS respectively (Dixon et al., 2012). Currently, the drugs and chemicals used to induce and inhibit ferroptosis in cells are as followed (Tables 1, 2).

**TABLE 1:** The ferroptosis inducers.

Affected pathways	Inducers	Mechanism	References
Iron metabolism or lipid peroxidation pathway	Artemisinin and its derivatives	Oxidize Fe2+ ions, promote ROS aggregation in cells and induce ferritin autophagy	(Chen et al., 2020a; Eling et al., 2015)
	MMRi62	Inhibit NCOA4 mutation and induce FTH1 degradation	Li et al. (2022a)
	FINO2	Direct oxidation of unstable iron and indirect inactivation of GPX	(Abrams et al., 2016)
	Siramesine with Lapatinib	Synergistic effect to induce ferroptosis by decreasing the expression of iron transporter and increasing the expression of transferrin, which leads to the increase of iron	Ma et al. (2016)
	iFSP	Inhibit FSP1 and sensitize cells to ferroptosis induced by RSL3	Doll et al. (2019)
	Iron-based nanomaterials	Specific targeting site, releasing Fe^2+^ or Fe^3+^ in acidic lysosome	Li et al. (2021a)
	PAB	Up-regulating transferrin receptor and activating NOX4	Wang et al. (2018)
Amino acid metabolic pathway	Erastin	Inhibit system Xc-, and prevent the intake of cystine; Inhibit VDAC 2/3 and affect mitochondrial function	(Dixon et al., 2012; Yang et al., 2020)
	Sulfasalazine	Reduce GSH by targeting Xc- transporter	Liu et al. (2021a)
	Sorafenib	Similar to erastin, it inhibits system Xc- and prevents cystine intake	Gao et al. (2021)
	RSL3	Covalently binding with GPX4	(Dixon et al., 2012; Tang et al., 2020)
	FIN56	Induce degradation of GPX4, bind and activate SQS, and reduce CoQ10	Shimada et al. (2016)
	Hexamethylmelamine	Inhibition of GPX4 activity	Ye et al. (2021)
	cis-platinum	Direct consumption of GSH	Guo et al. (2018)
	Piperis amide	GSH consumption	Yamaguchi et al. (2018)
Other metabolic pathways	Brequinar	Suppress DHODH	Mao et al. (2021)

**TABLE 2:** The ferroptosis inhibitors.

Affected pathways	Inhibitors	Mechanism	References
Iron metabolism	Ferrostatin-1	Eliminate unstable iron and ROS	Dixon et al. (2012)
	Curcumin	Suppressed erastin, regulated NRF2/HO-1 pathway	Yang et al. (2021)
lipid peroxidation pathway	Vitamin E	Suppressed LOX	Kagan et al. (2017)
	Baicalein	Regulated GPX4/ACSL4/ACSL3 axis, suppressed lipid peroxidation	Li et al. (2022b)
	Thiazolidinedione (TZD)	Selectively suppressed ACSL4	Doll et al. (2017)
	Lliproxstatin-1	Suppressed mitochondrial lipid peroxidation and reduced MDA levels and it upregulated GSH, GPX4 and FSP1	Fan et al. (2021)
	Zileuton	Specificly inhibited 5-LOX	Liu et al. (2015)
Amino acid metabolic pathway	BRD4770	Inhibition of the System Xc--GPX4 pathway	Chen et al. (2022)
Other metabolic pathways	Vitamin K	FSP1-dependent nonclassical vitamin K cycle	Mishima et al. (2022)

## The metabolic pathways of ferroptosis

Ferroptosis is mainly induced by iron metabolism, lipid metabolism, amino acid metabolism and other processes. Previous studies have shown that the ferroptosis process can be induced by the two antagonistic processes of lipid peroxide production and elimination within cells. Many metabolic pathways include cellular respiration (i.e. mitochondrial tricarboxylic acid cycle (TCA) and electron transfer chain, ETC), lipid metabolism and amino acid metabolism, which can lead to ferroptosis by producing reactive oxygen species (ROS). In addition, iron metabolism may also induce ferroptosis through Fenton reaction, which produces lipid peroxide (Battaglia et al., 2020; Lu et al., 2018). At present, metabolic pathways related to ferroptosis are shown below.

### Iron metabolism pathway

Iron is an indispensable trace element in life body, and free ferrous ions act as the leading role of ferroptosis. Excessive ferrous ions will undergo Fenton reaction and lead to ROS accumulation, which can be oxidized with polyunsaturated fatty acids (PUFA), resulting in massive accumulation of lipid peroxide, thus causing DNA damage. Ferrous ions often exist in ferritin to form unstable iron pools. When iron ions overload and exceed the buffering capacity of ferritin, ferritin autophagy will occur, which is mediated by nuclear receptor coactivation factor 4 (NCOA4) (Latunde-Dada, 2017). Over-expression of NOCA4 will increase concentration of intracellular free iron, then promote ferroptosis. Recent studies have shown that cytoplasmic iron chaperone poly (rC) binding protein 1 (PCBP1) can inhibit the ferroptosis process mediated by iron protein phagocytosis in head and neck cancer (Lee et al., 2022). Genes related to iron metabolism, such as transferrin (TF), transferrin receptor 1 (TFR1), iron transporter (FPN), divalent metal transporter 1 (DMT1), ferritin heavy chain 1(FTH1) and ferritin light chain (FTL), are key mediators in the process of ferroptosis. As an iron transporter, transferrin mediates iron uptake or iron autophagy, and introduces iron into cells from the extracellular environment by recognizing transferrin receptor 1 (TFR1), while down-regulating the expression of transferrin receptor (TFRC) can inhibit the occurrence of ferroptosis (Bogdan et al., 2016). Ferroportin-1(FPN) is an iron transporter responsible for removing iron from cells (Dixon et al., 2012; Ma et al., 2016). Recently, it has been reported that heat shock protein β-1 (HSPB1) reduces the intracellular iron concentration by inhibiting the expression of TRF1, thus inhibiting the ferroptosis process (Battaglia et al., 2020). In addition, heme is a source of iron ions within cells, and heme oxygenase-1 (HO-1) can decompose heme and release iron ions, and regulate the process of ferroptosis (Kwon et al., 2015). Nuclear factor-erythroid 2-related factor 2 (Nrf2) dissociates from Keap1 when receiving external stimulation, transfers to the nucleus, binds to the promoter region, and activates the downstream molecule HO-1 (Dodson et al., 2019).

### Lipid metabolism pathway

As for inducing cell damage, lipid peroxide can be regarded as the “executor” of ferroptosis. Lipid peroxide causes cell damage through the following ways: First, the lipid peroxide produced by Fenton reaction reacts with ferrous iron to produce ROS, which aggravates the ferroptosis cycle; Second, 4-HNE and MDA, the aldehyde degradation products of lipid peroxide, generate cytotoxicity; Last, peroxide reaction interferes with the permeability and fluidity of cell membrane (Gaschler and Stockwell, 2017). Among them, the activation of cyclooxygenase (COX), lipoxygenase (LOX), acyl-CoA synthetase long chain family member 4 (ACSL4) and lysophosphatidylcholine acyltransferase 3 (LPCAT3) play an important role in lipid oxidation and anabolism (Liu et al., 2022). ACSL4 promotes the formation of phytosterol ester esterified by arachidonic acid (AA) and adrenaline, and participates in the ferroptosis process. According to the research, the tumor suppressor miR-424-5p directly binds to the 3′-UTR of ACSL4 to inhibit the process of ferroptosis (Cheng et al., 2020). Vitamin E acts as a potent inhibitor of ferroptosis by inhibiting LOX activity (Kagan et al., 2017).

### Amino acid metabolic pathway

Cystine/glutamic acid reverse transporter (system Xc-) is composed of light chain subunits (xCT,SLC7A11) and heavy chain subunits (CD98hc,SLC3A2), of which SLC7A11 is the active part. System Xc- mediates extracellular cystine to enter cells to synthesize glutathione (GSH). GSH can be converted into oxidized glutathione (GSSG) *via* GPX4 (Glutathione peroxidase 4), restoring active oxygen and active nitrogen, thus limiting the spread of lipid peroxidation in the membrane and alleviating the oxidative stress damage caused by ferroptosis (Dixon et al., 2012). GPX4 is a key inhibitor of ferroptosis and constitutes the defense system of ferroptosis. There are reports that RAS selective lethal small molecule 3 (RSL3) binds to GPX4 and inhibits its activity (Dixon et al., 2012; Yang et al., 2014). Erastin can target system Xc and selectively induce ferroptosis (Wang et al., 2020). Therefore, the inhibition of Systeam Xc-, the blocking of GSH synthesis and the inactivation of GPX4 may serve as an important target for inducing ferroptosis in tumor cells.

### Other metabolic pathways

The inhibition of ferroptosis by ferroptosis inhibitor protein (FSP1) is mediated by ubiquitin (also known as coenzyme Q10, CoQ10). The reduced form of ubiquitin can capture lipid peroxidation free radicals, while FSP1 catalyzes the regeneration of CoQ10 through NAD(P)H. The FSP1-CoQ10-NAD(P)H pathway acts as an independent parallel system, which collaboratively inhibits ferroptosis together with GPX4 and glutathione (Doll et al., 2019). In addition, recent studies have confirmed GTP cyclohydrolase I (GCH1) to be another important regulator of ferroptosis.

## Ferroptosis regulation in different cancers

In the process of tumor occurrence and development, it is often accompanied by the imbalance of redox environment and the high demand for iron ions, suggesting that tumor cells are highly sensitive to iron death. We have discussed the metabolic pathway, the application of inducers and inhibitors of ferroptosis, then the regulation of ferroptosis in multiple cancers will be introduced.

Ovarian cancer is a highly malignant tumor, which usually exhibit a high demand for iron. It is manifested by up-regulation of transferrin receptor and down-regulation of membrane iron transporter and ferritin, suggesting its high sensitivity to ferroptosis. The synergistic effect of Erastion and various chemotherapeutic drugs can overcome the drug resistance in ovarian cancer cells (Li et al., 2021b). According to recent studies, ferroptosis is considered to mediate the efficacy of Olapani (a classic and effective PARP inhibitor) *in vitro* and *in vivo*. Researchers believe that pharmacological inhibition of PARP or gene deletion promotes lipid peroxidation and ferroptosis in ovarian cancer cells. The efficacy and safety of the triple therapy of Olapani, FINs and DNA damage induction in BRCA wild-type ovarian cancer deserve further exploration (Hong et al., 2021).

Studies have confirmed that ferroptosis occurs when liver cancer cells are treated with sorafenib. Sigma-1 receptor (S1R) is an important negative regulator of ferroptosis in liver cancer cells, and regulates many targets of iron death, such as GPX4 (Bai et al., 2019). In addition, the activation of P62-Keap1-NRF2 antioxidant pathway can resist ferroptosis in liver cancer cells, and the inhibition of this pathway significantly enhances the anticancer activities of erastin and sorafenib *in vitro* and *in vivo* (Sun et al., 2016).

In the treatment of advanced gastric cancer, cisplatin and paclitaxel, as clinical first-line chemotherapy drugs, have increasingly serious drug resistance, and the targeted ferroptosis pathway has brought a new dawn. Arachidonic acid lipoxygenase-15 (ALOX-15) is the main producer of ROS in cells, which is obviously down-regulated in gastric cancer cells and is found to inhibit ferroptosis. While miR-522 secreted by tumor-associated fibroblasts (CAFs) regulates the expression of ALOX15, it can provide a new way to improve the sensitivity of chemotherapy in exploring the mechanism of regulating ferroptosis by exosomes (Zhang et al., 2020).

Studies have shown that the activity of polyunsaturated lipid synthase increases when colorectal cancer cells are in a highly interstitial state, indicating the potential link between interstitial state and lipid peroxidase pathway (Viswanathan et al., 2017). In addition, cetuximab is prone to drug resistance in the treatment of colon cancer, while its combination with ferroptosis inducer β-elemene can sensitize KRAS mutant colorectal cancer cells *via* inducing ferroptosis and inhibiting epithelial-mesenchymal transition (EMT) and is expected to provide a prospective treatment strategy for CRC patients with RAS mutant (Chen et al., 2020b).

At first, Doxin et al. found small molecules that can specifically kill RAS mutant cancer cells through ferroptosis, such as erastin and RSL3. Coincidentally, about 90% of pancreatic vessel element’s carcinoma (PDAC) has KRAS gene mutation (Mizrahi et al., 2020). The artemisinin and its derivatives can promote the occurrence of ferroptosis in PDAC by inducing ROS production (Eling et al., 2015). In addition, HSPA5 inhibits ferroptosis by directly inhibiting the degradation of GPX4 protein in human PDAC cells, and the up-regulation of HSPA5-GPX4 pathway contributes to the drug resistance of gemcitabine. And the inhibition of ATF4-HSPA5 pathway can enhance the ferroptosis induced by erastin (Zhu et al., 2017).

As the nervous system contains the highest content of polyunsaturated fatty acids in human body, which is the main substrate for peroxide production, ferroptosis in brain tumors has gradually attracted attention. The temozolomide (TMZ) has been applied in the treatment of glioblastoma (GBM), and TMZ can selectively induce glioma stem cells (GSCs) to ferroptosis during treatment (Xu et al., 2019).

## Ferroptosis and immunotherapy

Immunotherapy can act on the immune system through “passive” or “active” therapy. Passive therapy includes the application of cytokines, antibodies and immune cells, which directly acts on the immune cells within tumor micro-environment. Active therapy involves stimulating the immune system to eliminate cancer cells (such as vaccines) (Peng et al., 2019). Up to now, anti-tumor immunotherapy includes the following means.

### Nonspecific immunotherapy

Cytokines are protein secreted by immune cells and other cells, which restrict the tumor growth through direct anti-proliferation or pro-apoptosis activity or stimulating the cytotoxic activity of immune cells to tumor cells (Berraondo et al., 2019). Cytokines include tumor necrosis factor (TNF)-α, interferon (IFN) and IL-2, etc. IL-2 is a T cell growth factor, which participates in the growth and expansion of immune cells such as T cells, NK cells and B cells. It is one of the most widely studied cytokines and immunotherapy agents, and is used to treat cancers and other diseases. At present, IL-2 has been approved by FDA to treat melanoma and renal cell carcinoma. However, due to the serious systemic toxicity and limited curative effect of IL-2, researchers turned to explore optimized schemes, such as combination therapy. There are studies that combined treatment of aerosol IL-2 and NK cells can enhance the therapeutic effect in lung metastatic tumors (Dhupkar and Gordon, 2017).

### Antibody therapy

Antibody therapy is the most mature cancer-specific immunotherapy in clinical practice at present. Rituximab is the first monoclonal antibody approved by FDA for anti-tumor immunotherapy, which targets CD20 antigen and is used to treat B-cell non-Hodgkin’s lymphoma (Sanchez-Martin et al., 2015). With the development of research, besides directly targeting tumor surface antigen, targeting key signal pathway of tumor to diminish tumor growth environment and immune checkpoint blocking therapy are also being under road (Helmy et al., 2013). As for immune checkpoint blocking therapy, the current breakthrough is mainly the recognition and targeting role in T cells by antibodies of CTLA-4, PD-1 or PD-L1 (van den Bulk et al., 2018).

### Adoptive immune cell therapy

The main principle of adoptive cell transfer therapy (ACT) is to inject immune cells into the patient’s body after *in vivo* immunization or *in vitro* culture activation, so as to enhance the patient’s immunity. At present, ACT is mostly carried out in hematological malignancies, while CAR-T cell technology has also been conducted in sarcoma targeting ERBB2/HER2, renal cell carcinoma targeting carbonic anhydrase, and cholangiocarcinoma targeting epidermal growth factor (Rohaan et al., 2019).

### Ferroptosis-related genes

The biomarker of ferroptosis is of great significance for predicting curative effect and realizing subsequent precise molecular targeted therapy. Ferroptosis-related genes (FRGs) can be divided into genes about iron metabolism, lipid metabolism, oxidant metabolism and energy metabolism. Recent studies have shown that FRGs features can predict the prognosis in various kinds of tumors (Wan et al., 2021; Wang et al., 2021). For example, when constructing the characteristic risk model of ferroptosis in glioma, most selected FRGs were differentially expressed in patients with different pathological grades and non-tumor control groups. It is reported that in the prognosis model of hepatocellular carcinoma, the genes (NFS1, CISD1, ACSL3, NQO1, SLC7A11, GPX4) that can protect hepatocytes with ferroptosis and the genes (ACACA, CARS, G6PD, SLC1A5) that induce ferroptosis are all up-regulated in HCC, and are related to poor prognosis (Liang et al., 2020). This suggests that constructing a gene characteristic model related to ferroptosis has shown predictive value for tumor prognosis.

In the model of ferroptosis-related gene features, there is positive correlation with the enrichment scores of ICB-related positive features in the high-risk scoring group. In addition, researchers observed that immune signals were related to different risk scores of glioma, showing increase of PD-L1, PD-1, CTLA-4, IDO-1, TMB and immunogenic mutations in high-risk scoring groups (Wan et al., 2021). In the model constructed in breast cancer, the antigen presentation level and Th1 cell level of high-risk population are elevated, suggesting significant change of related genes, which may be related to the over-activation of immune system (Wang et al., 2021). Generally, these studies indicate that the development of FRGs-related risk score may help predict the anti-tumor effect of immunotherapy.

### Effect of ferroptosis on tumor microenvironment

Tumors usually show low immunogenicity to escape the recognition of immune cells. Ferroptosis-related lipid peroxide can recognize dendritic cells, phagocytosis and processing of tumor antigens, and present tumor-related antigens to CD8^+^ lymphocytes, activating cytotoxic T lymphocytes to aid anti-tumor immunotherapy (Zhao et al., 2022). Ferroptosis plays an important role in the activity and function of tumor-associated macrophages (TAM) in TME. TAM mainly exhibits M2 subtype, which inhibits anti-tumor immunity. However, M1 subtype with higher anti-tumor activity showed higher resistance to ferroptosis caused by deletion of GPX4. There is evidence that targeting GPX4 in TAM can inhibit the survival of M2 subtype, while keeping the number of M1 unaffected, thus reversing the immunosuppressive state (Xu et al., 2021a). At the same time, some studies have found that ferroptosis promotes the release of Kras^G12D^, which contributes to M2 polarization and stimulates tumor growth through STAT3-dependent fatty acid oxidation pathway (Liu et al., 2021b). Therefore, the dual effects of ferroptosis on TAM need to be further studied. T-reg cells are thought to impair anti-tumor immunity. Although ferroptosis is rare in T-reg cells, recent studies have shown that targeting GPX4 can disturb the immune homeostasis, promote the production of IL-1β and Th17 cell reaction, thus enhancing the anti-tumor immune function (Xu et al., 2021b).

### Combined application of ferroptosis with immunotherapy

Recently, it has been found that CD8+T cells can enhance lipid peroxidation caused by ferroptosis in tumor cells. Meanwhile, ferroptosis improves the anti-tumor effect of immunotherapy (Wang et al., 2019). This suggests a novel therapeutic strategy. CD8+T cells activated by anti-PD-L1 immunotherapy secrete IFN-γ, which significantly down-regulates the expression of SLC3A2 and SLC7A11 in tumor cells, resulting in the decrease of cystine uptake, thus promoting the occurrence of ferroptosis. The results showed that cystine protease inhibitor could cooperate with anti-PD-L1 to induce effective anti-tumor immunity through ferroptosis (Wang et al., 2019). Based on the development of immunogenic cell death (ICD) in anti-cancer experiments, researchers have found that many cancers can produce necrotic apoptosis resistance. Recently, the research on the immunogenicity of ferroptosis made rapid progress. Cancer cells with early ferroptosis can effectively induce ICD *in vivo* and *in vitro* by activating bone marrow-derived dendritic cells BMDCs and triggering DAMP (e.g. ATP and HMGB1), resulting in protective immunity against the attack on cancer cells, which boost the development of anti-cancer immunotherapy strategies based on ferroptosis cell vaccination (Efimova et al., 2020). Recent studies have shown that RSL-3-rich nanoparticles can promote the immunogenic death of tumor cells, while the combined treatment of blocking PD-L1 further enhanced the T lymphocytes infiltration within tumors (Song et al., 2021).

Studies have shown that the synergistic effect of radiotherapy and immunotherapy is related to the increased sensitivity to ferroptosis. The anti-tumor effect of radiotherapy is not only related to DNA damage, but also lipid peroxidation caused by ferroptosis. Radiotherapy and immunotherapy can induce ferroptosis of tumor cells by activating kinases ATM and IFN-γ through DNA damage, respectively (Lang et al., 2019). In addition, inhibiting SLC7A11 or GPX4 combined with FINs can significantly improve the curative effect of radiotherapy and reverse radiation resistance (Lei et al., 2020). For those tumors that show anti-ferroptosis characteristics, combining FINs with immunotherapy and radiotherapy may be an effective strategy to sensitize such tumors. However, whether this triple therapy increases the toxicity of normal tissues remains to be determined.

In order to overcome the drug resistance of immunotherapy, researchers have focused on the emerging target of ferroptosis. Studies have shown that the expression of TYRO3 is increased in immunotherapy-resistant tumor cells, and TYRO3 signaling pathway inhibits the ferroptosis of tumor cells by up-regulating the expression of SLC3A2 and other genes. TYRO3 receptor tyrosine kinase inhibitors can effectively overcome the drug resistance of immunotherapy (Jiang et al., 2021). As mentioned above, the combination of β-elemene and cetuximab, an ferroptosis inducer, can induce ferroptosis in the colorectal cancer cell with KRAS mutation, thus relieving the drug resistance caused by cetuximab in CRC treatment (Chen et al., 2020a). In addition to inducing ferroptosis in tumor cells, ferroptosis will also occur in T cells themselves. Recent studies have shown that CD36-mediated ferroptosis of T cells inhibits the function of CD8+T cells in tumors. The combination of CD36 deletion and anti-PD-1 antibody has a better anti-tumor effect than single treatment (Ma et al., 2021). Therefore, targeting CD36 and ferroptosis may be an effective strategy to improve the anti-tumor efficacy of T cell-based immunotherapy.

In a word, different tumor cells exhibit differential sensitivity to immunotherapy and the combined therapeutic effect with ferroptosis on tumor cells is distinct. In immunoinflammatory tumors with a large number of CD8+T cells, ferroptosis inducers may diminish infiltrated T cells, weaken the maturation process and normal function of immature dendritic cells (DC), and thus reduce the efficacy of ICI immunotherapy. In some cases, ferroptosis inhibitors may be utilized to protect T cells from ferroptosis caused by specific TME. For immune-tolerant tumors, there are high levels of infiltrating myeloid-derived suppressor cells (MDSCs), tumor-associated macrophages (TAMs) or Tregs in TME. GPX4 inhibitor can eliminate M2 macrophages and Treg, while targeting system Xc- can alleviate MDSC-mediated uptake of tmecystine, thus ensuring the survival of T cells. However, for some immune-desert tumors lacking immune infiltration, the response to immunotherapy is poor, and chemotherapy and targeted drugs are used to counteract the resistance. Therefore, more attention should be paied to the research of ferroptosis in improving anti-tumor immunogenicity (Xu et al., 2021a).

### Comparison with other forms of cell death

In recent years, tumor immunotherapy represented by immune checkpoint inhibitors (ICIs) has received a lot of attention. However, it has little effect on some immune-desert tumors, and the application of ferroptosis may change TME state and enhance the therapeutic effect (Rosenbaum et al., 2021). The main mechanism and application progress of other cell death forms in combined immune checkpoint therapy go as follows (Table 3).

**TABLE 3:** Comparison of ferroptosis with other forms of cell death in immunotherapy.

Death form	Immune characteristics	Combined blocking of PD-1/PD-L1 immune checkpoint	Other immune checkpoint combination therapy	Application	References
Autophagy	Usually anti-inflammatory	HIP1R can induce PD-L1 degradation in lysosomes, activate T cells and inhibit tumor growth	Increase the expression of CTLA-4, restore the activity of CTLA-4 inhibitor, act on Tregs, and inhibit inflammation and inflammatory cancer	breast cancer	Gao et al. (2021)
Pyroptosis	pro-inflammatory	The N-terminal of GSDMA3 can induce pyroptosis and release IL-18, and increase the inhibition of tumor cells mediated by T cells. PD-L1 can regulate GSDMC. Gmd and GSDMD can be used as biomarkers for anti-PD-1 therapy	RIG-I agonist can mediate cell apoptosis by activating caspase-1 and GSDMD, and DPP8/9 may be a new immune checkpoint for activating anti-tumor immune system	breast cancer	Li et al. (2021c)
Necrotic apoptosis	pro-inflammatory	Trigger type I IFN reaction, reshape the cross-start and proliferation of TME and CD8^+^ T cells, and enhance the anti-tumor immunogenicity		melanoma, squamous cell carcinoma of neck	Wang et al. (2022)

## Summary

As a new way of cell death, ferroptosis is related to many physiological and pathological mechanisms, and become a hot spot in cancer treatment research. In addition, many tumors especially those with low levels of tumor infiltrating lymphocytes (TIL), are called “cold” tumors, and have poor response to ICI. Therefore, it is vital to develop new strategy to enhance anti-tumor immunity. Moreover, the defense mechanism of iron death in cells (especially mitochondria) has also been reported, which indicates the limitation and breakthrough of ferroptosis in immunotherapy (Battaglia et al., 2020). As the ferroptosis inducers/inhibitors have been widely illustrated and applied in pre-clinical study, the combined application of ferroptosis induction with tumor immunotherapy (especially ICI therapy) has shown promising prospective in clinical treatment. However, there are still some limitations of ferroptosis in immunotherapy, for example, the dual role of anti-/pro- immunity in ferroptosis, the different immune characteristics of tumor cells, and the complexity of TME all enhance the application difficulty, and more research should be conducted in the future.
